# Decreasing Risk of Hepatitis A Infection in León, Nicaragua: Evidence from Cross-Sectional and Longitudinal Seroepidemiology Studies

**DOI:** 10.1371/journal.pone.0087643

**Published:** 2014-02-11

**Authors:** Orlando Mayorga Perez, Martin W. G. Brinkhof, Matthias Egger, Gert Frösner, Christian Herzog, Marcel Zwahlen

**Affiliations:** 1 Department of Microbiology and Parasitology, Faculty of Medical Sciences, National Autonomous University, León, Nicaragua; 2 Institute of Social & Preventive Medicine (ISPM), University of Berne, Berne, Switzerland; 3 Max von Pettenkofer Institute, Ludwig-Maximilians University, Munich, Germany; 4 Crucell Switzerland AG, Berne, Switzerland; Kaohsiung Medical University Hospital, Taiwan

## Abstract

**Background and Objectives:**

Nicaragua is highly endemic for hepatitis A. We aimed to provide an estimate of the change in the age-specific risk of hepatitis A virus (HAV) infection based on serological data from cross-sectional and longitudinal samples collected in León, Nicaragua, in 1995/96 (n = 979) and 2003 (n = 494).

**Methods:**

The observed age-specific prevalence of anti-HAV antibodies was correlated to the age-specific risk of infection by calculating the probability of freedom from infection at a specific age.

**Results:**

The proportion of seropositive children aged 1.5 to 6 years was 42% in 2003 compared to 67% in 1995/96. Estimated annual risk of infection for a 3-year old child was 30% (95% CI: 27.0%, 33.1%) in 1995 and 15.5% (95% CI: 12.4%, 19.0%) in 2003. There was good agreement between estimates based on cross-sectional and longitudinal data. The age-specific geometric mean of the quantified anti-HAV antibody levels assessed in 2003 was highest at age 4 and decreased steadily up to age 40.

**Conclusions:**

The substantially lower risk of HAV infection in 2003 than in 1995 for young children indicates a beginning transition from high to intermediate endemicity in León, Nicaragua. Consecutive age-stratified serosurveys are useful to assess changes in risk of infection following public health interventions. The decreasing age-specific GMC of anti-HAV antibodies during adulthood in a country with endemic HAV indirectly suggests that ongoing HAV exposure in the community has marginal boosting effect on antibody levels once protective immunity has been established by natural infection.

## Introduction

An estimated 212 million cases of hepatitis A virus (HAV) infection [1] and 31 million cases of symptomatic illness (S.Wiersma, personal communication) occurred worldwide in 2005, with some 35,000 estimated deaths [1]. The clinical presentation depends on the age at infection. In children younger than 6 years, hepatitis A is usually asymptomatic, while most adolescents or adults develop moderate to severe symptoms and jaundice [1], [2]. Because HAV infection confers life-long immunity, seroprevalence rates in different age groups are indicators of susceptibility to infection in a population [2], [3]. In areas of high endemicity, with a life-time risk of infection above 90% [1], most HAV infections occur in early childhood and clinical symptoms of hepatitis A are rarely seen. In areas of low endemicity, mainly adolescents and adults are infected. It follows that epidemics of clinically manifest HAV infection are uncommon in highly endemic areas whereas in areas with intermediate endemicity, hepatitis A infections occur primarily as outbreaks [4].

Universal childhood hepatitis A vaccination is an effective means to control HAV infections in a given population, whereby - due to herd immunity - the morbidity not only of the target toddler population but also of the older children and of the adult population declines to low levels within a few years [5], [6]. Improving socioeconomic conditions in developing countries alone result in a transition from high to intermediate endemicity, with an increased number of susceptible individuals at higher ages [2], [3], [7]. The understanding of such transitions in conjunction with the shift in age-related risk of infection is crucial in predicting HAV transmission patterns and for decisions on appropriate prevention strategies [1], [8], [9].

Nicaragua is a country with high endemicity and low burden of clinical HAV as most infections are asymptomatic [10]. The aim of the present analysis was to provide an estimate on the changes in the age-specific risk of hepatitis A infection, based on age-specific seroprevalence data collected in León, Nicaragua, in 1995 and 2003. We show that the conduct of repeated cross-sectional surveys providing estimates of age-specific HAV antibody prevalence can be used to estimate the force of infection in a given population and might be used to monitor public health interventions.

## Methods

### Study populations and data sources

Seroprevalence of anti-HAV antibodies was determined from analyses of samples collected in two studies conducted in 1995/96 and 2003, respectively, in the same deprived area of Nicaragua's second city León. The first study was a placebo-controlled, randomized, field efficacy study of a virosome hepatitis A vaccine in children aged 1.5 to 6 years [10]. Serum samples were collected from all screened children (cross-sectional sample 1995). Longitudinal serological data from the seronegative children enrolled into the placebo group of the field trial were also analyzed, with venous blood drawn at week 6 and at months 3, 6, 9, 12, 15, and 18 (longitudinal sample 1995/96) [10]. In the 2003 study, serum samples were collected from individuals aged 0.5 to 40 years enrolled into a population-based serosurvey (cross-sectional sample 2003). In both cross-sectional studies data on age and sex had been collected. Data on sanitary and socio-economic conditions (number of household members, number of rooms, availability of indoor water and flush toilet) were collected upon enrollment into the 1995/96 longitudinal and the 2003 cross-sectional study using a standardized questionnaire.

### Ethics Statement

Both studies had been performed in accordance with the Declaration of Helsinki and Good Clinical Practice and the study protocols had been approved by the local ethics committee (Comité de Ética para investigaciones biomedicas (CEIB), Universidad Nacional Autónoma de Nicaragua (UNAN), Facultad de Ciencias Médicas, León). Written informed consent was obtained prior to any study related activities from the parents or guardians on the behalf of the minors and children participating in the 1995/96 hepatitis A vaccine field efficacy study and the 2003 serosurvey and from the adult subjects themselves in the 2003 serosurvey. The ethics committee had approved the consent procedure.

### Laboratory tests

In the seroepidemiology study 2003 anti-HAV antibodies were quantified with the microparticle enzyme immunoassay HAVAB 2.0 Quantitative of the AXSYM system (Abbott Diagnostics Division, Wiesbaden, Germany), with a lower limit of detection of ≥10 mIU/mL. For the purpose of screening (field study 1995/96) only qualitative analyses were carried out using the Enzygnost Anti HAV test (Behring, Marburg, Germany) with a cut-off level of ≥20 mIU/mL, as described earlier [10]. Both immunoassays used the WHO standard anti-HAV serum as reference.

### Statistical methods

In addition to seroprevalence rates in the 1995/96 and 2003 samples, the age group-specific geometric mean antibody concentrations (GMC) and minimum and maximum anti-HAV antibody levels were extracted from the cross-sectional serology data of the 2003 survey. GMCs were calculated from the log-transformed antibody concentration values of the anti-HAV antibody positive individuals for the various age groups.

In the analysis of the 1995/96 and 2003 cross-sectional prevalence data children <1 year old were excluded because their antibody results (low levels of 12–653 mIU/ml) do not represent the cumulative risk of infection in the first year of life but, reflect – as documented with other infant data from León [11] - the presence of maternal antibodies. As described in detail by others [12], [13], [14], the observed age-specific prevalence of anti-HAV antibodies is a function of the age-specific annual risk of infection. Similar to survival analysis, the relationship can be expressed as the probability to remain free of infection up to a given age A. For example, the probability to remain free of infection in the second year of life is equal to 1 minus the annual risk of infection in the second year of life (denoted as riskage(1 to 2)). The formula is as follows:
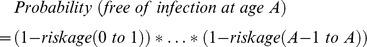



In a next step, we allowed the risk of infection to vary by age, sex and time of survey. This was done by modeling the logit of the annual risk of infection as a function of age (quadratic), sex (an indicator variable for male versus female), and calendar year (an indicator variable for 1995 vs. 2003) [12], [13], [14]. The model was fitted to the cross-sectional seroprevalence data of the years 1995 and 2003 to obtain estimates of the age-specific risk of infection using Markov chain Monte Carlo methods implemented in WinBUGS 1.4.

Serological data from the longitudinal sample were used to obtain direct estimates of the age-specific risk of HAV infection by splitting of the follow-up time until seroconversion for each subject over age strata. Direct estimates of annual infection risk were compared to those from the cross-sectional samples.

## Results

The two cross-sectional serosurveys consisted of samples collected in 1995 from 979 children aged 1.5 to <7 years screened for the vaccine field trial [10] and of 496 individuals (including 231 children aged 1 to <7 years) who took part in the 2003 serosurvey. The longitudinal sample comprised consecutive 3-monthly sera from 117 children aged 1.3 to 5.9 years who in the course of the field trial 1995/96 [10] had received placebo. Demographic and socio-economic data are shown in Table 1. Groups were balanced with respect to sex and the availability of flush toilets. Crowding and the availability of indoor water were somewhat lower in the 2003 cross-sectional compared to the 1995/1996 longitudinal study. None of the participants enrolled and tested in the 2003 serosurvey had been vaccinated against hepatitis A.

**Table 1:** Socio-demographic data of individuals included in the cross-sectional and longitudinal samples.

		1995	1995/96	2003
		cross-sectional	longitudinal	cross-sectional
Total number of subjects included		974*	117	496**
Sex, No (%)	male	496 (50.9)	60 (51.3)	244 (49.2)
	female	478 (49.1)	57 (48.7)	252 (50.8)
No by age (mean age; range)	1–<7 years	974 (3.42; 1.4–6.1)	117 (2.95; 1.3–5.9)	231 (3.1; 1.0–6.0)
	7–11 years	n.a.	n.a.	37 (9.6; 7.2–11.9)
	12–16 years	n.a.	n.a.	33 (14.1; 12.0–16.2)
	18–25 years	n.a.	n.a.	56 (22.1; 17.9–25.7)
	26–33 years	n.a.	n.a.	56 (30.2; 26.1–33.8)
	34–40 years	n.a.	n.a.	53 (38.4; 34.0–40.7)
No of inhabitants per room (mean ± sd)***	1–<7 years	n.a.	4.0±2.3	3.6±2.1
Indoor water (%)***	1–<7 years	n.a.	106 (90.6)	187 (81.0)
Flush toilet (%)***	1–<7 years	n.a.	42 (35.9)	77 (33.3)

n.a. = not applicable or not assessed.

* For 5 out of 979 children enrolled (field trial screening^12^) the exact age was not known.

** Includes 30 infants (<1 year of age).

*** In households with children 1–<7 years.

### Seroprevalence

The age-specific seroprevalence of naturally acquired anti-HAV antibodies is shown in Table 2. Seroprevalence rates and risk of infection were lower in the population sampled in 2003 than in children surveyed in 1995. In 1995, 67% of children aged 1.5 to <7 years were anti-HAV positive with the proportion of seropositive children increasing from 51% at the age of 2 years to 87% at the age of 6 years. In 2003, seroprevalence in the same age group overall was 42% and the proportion of seropositive children increased from 23% at the age of 2 years to 62% at the age of 6 years.

**Table 2:** Seroprevalence of naturally acquired anti-HAV antibodies in the cross-sectional samples from 1995 and 2003.

	Seroprevalence n/N (%)	
Age groups	1995* (n = 974)	2003** (n = 494***)
6–11 months	n.a.	14/30 (46.7) #
12–17 months	n.a.	4/34 (11.8) ø
18–23 months	48/126 (38.1)	3/28 (10.7)
24–29 months	58/113 (51.3)	7/29 (24.1)
30–35 months	83/145 (57.3)	12/35 (34.3)
36–47 months	170/232 (73.3)	15/33 (45.5)
48–59 months	150/189 (79.4)	23/38 (60.5)
60–71 months	148/169 (87.6)	20/32 (62.5)
7–11 years	n.a.	32/37 (86.5)
12–16 years	n.a.	32/34 (94.1)
18–25 years	n.a.	56/56 (100)
26–33 years	n.a.	56/56 (100)
34–40 years	n.a.	53/53 (100)

* ≥20 mIU/mL anti-HAV antibodies (ELISA Enzygnost Anti HAV Behring).

** ≥10 mIU/mL anti-HAV antibodies (HAVAB 2.0 quantitative EIA AxSYM Abbott).

*** For two children (age group 18–23 months) no anti-HAV data were available.

# All 14 children had maternal anti-HAV antibodies (range; 12–653 mIU/mL).

ø 1 child of 4 children had recent HAV infection (13700 mIU/mL), 3 children had maternal antibodies (26, 28, and 31 mIU/mL).

n.a. = not applicable.

### Annual risk of HAV infection

**Figure 1:**
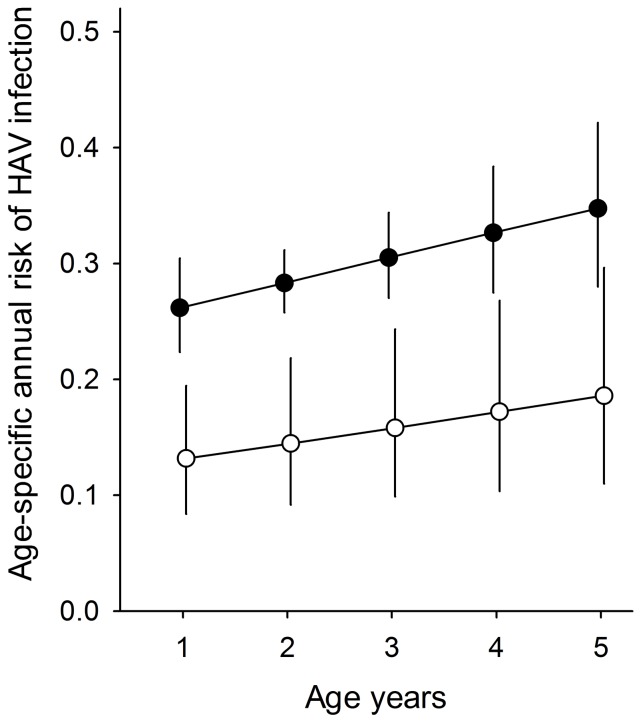
Estimated age-specific risk (95% confidence intervals) of HAV infection based on cross-sectional samples. Solid circles represent estimates derived from the 1995 survey; open circles are estimates from the 2003 data.

The estimated annual risk of infection for a 3-year old child was 30% (95% CI 27.0% to 33.1%) in 1995 compared with 15.5% (95% CI 12.4% to 19.0%) in 2003 (Figure 1). There was no difference between female and male sex in the risk of infection (adjusted odds ratio [OR] male vs. female = 1.0 [95% CI 0.87 to 1.18]). However, calendar year had a significant effect, with a lower risk of infection for children in the 2003 cohort (OR 2003 vs. 1995 = 0.43 [95% CI 0.32 to 0.54]).

**Figure 2:**
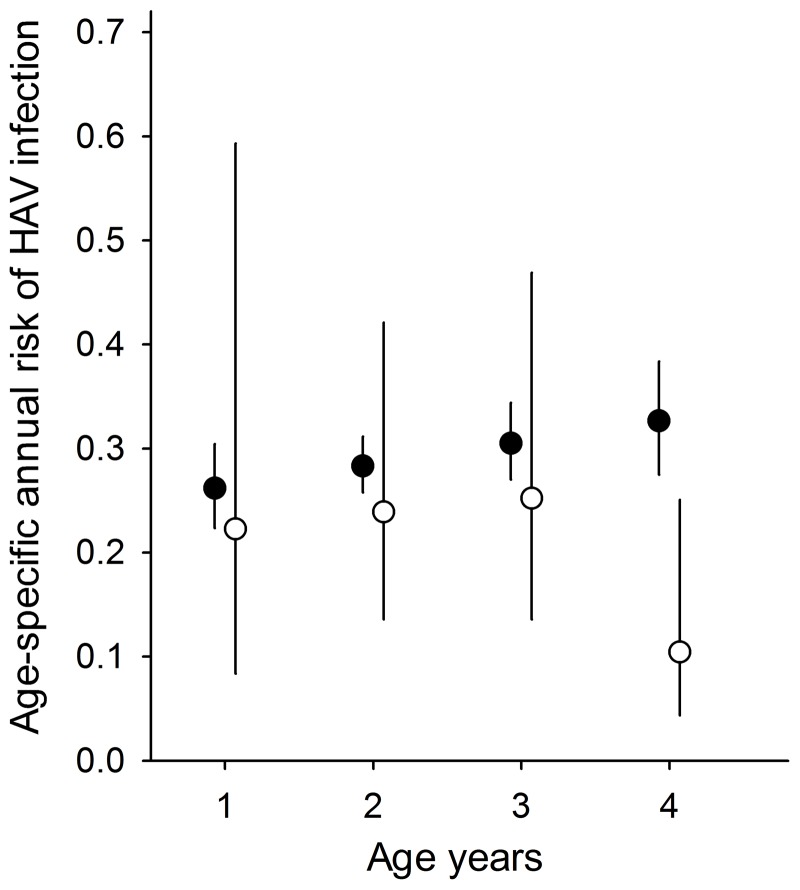
Estimated age-specific risk (95% confidence intervals) of HAV infection in 1995 from cross-sectional and longitudinal samples. The solid circles represent estimates derived from the cross-sectional 1995 survey and open circles the estimates from the longitudinal data.

Analysis of the longitudinal data obtained from the placebo group of the 1995 study showed an annual 25% (95% CI 14% to 46%) risk of infection for a 3-year old child (Figure 2). There was good agreement between the estimated annual risk of infection from the longitudinal and cross-sectional studies in the 1995/96 samples, with the exception of 4-year old children in whom the longitudinal data indicated a lower risk of infection compared with that calculated from the cross-sectional sample (Figure 2).

### Quantitative anti-HAV levels

**Figure 3:**
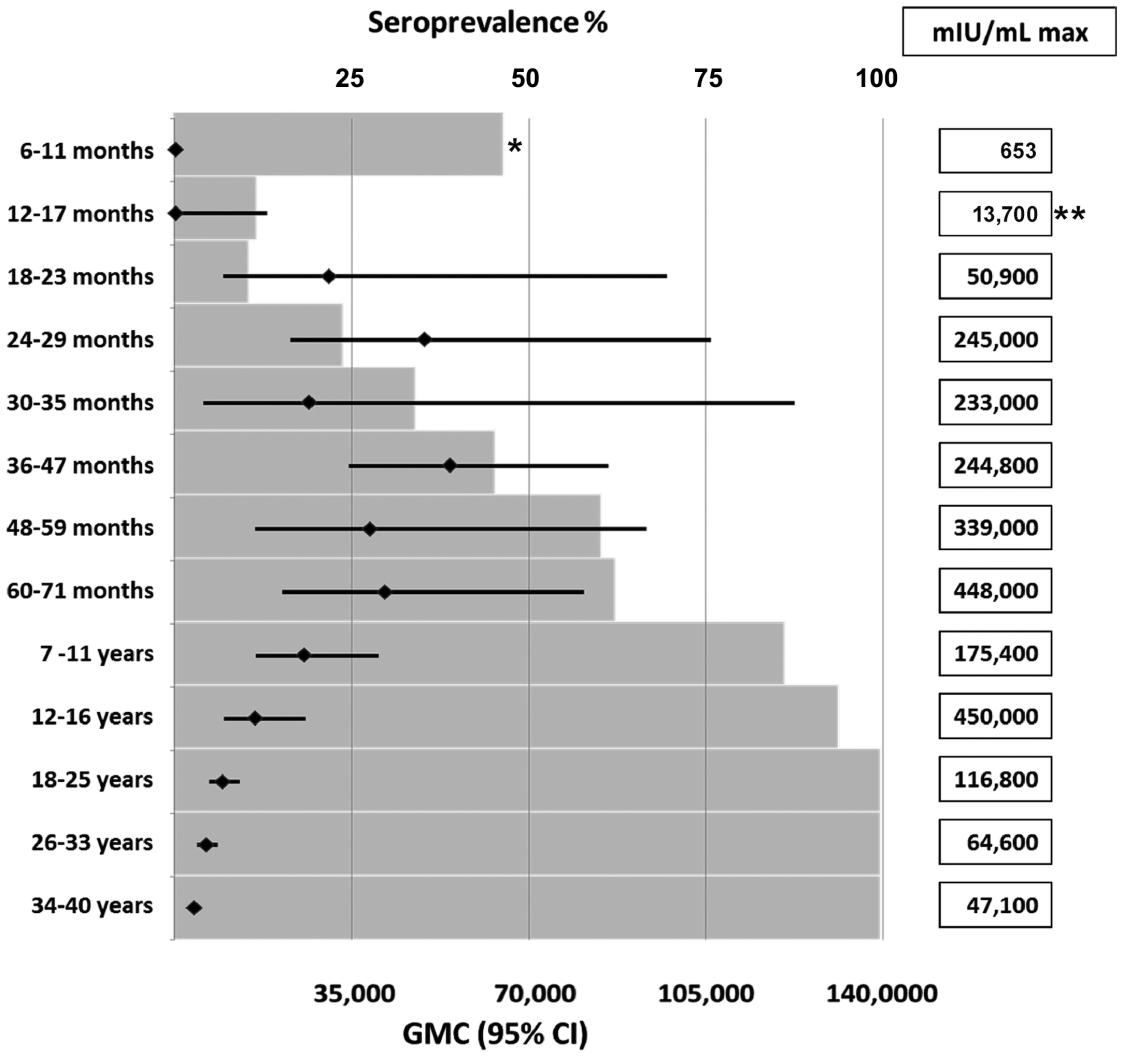
Age-specific seroprevalence, GMC and maximum values of anti-HAV antibodies in the 2003 cross-sectional sample. The shaded bars (upper scale) represent seroprevalence rates; the diamonds (lower scale) indicate GMC with lines representing 95% confidence intervals. * The seroprevalence of the 6–11 months old children is entirely due to maternal anti-HAV antibodies (see Table 2). ** The maximal concentration of 13700 mIU/mL was caused by one recent HAV infection. Three other anti-HAV positive children in this group had maternal anti-HAV with concentrations of 26, 28 and 31 mIU/ml (see Table 2).

Figure 3 shows for the 2003 serosurvey the age-specific seroprevalence in age groups ranging from 6 to 11 months up to 34 to 40 years along with the GMC values and maximum values of anti-HAV antibodies. In the younger age groups, seroprevalence and GMC increased first in parallel, with GMC values leveling off at age 4 to 5 years and then declining steadily until age 34 to 40 years. From the age group 12 to 16 years onwards seroprevalence reached 94% and then remained at 100%. Very high maximal concentrations of up to 450,000 mU/mL were recorded in children and adolescents aged 5 to 16 years.

## Discussion

In Nicaragua and many other low-income and middle-income countries, public health-measures to reduce intestinal and respiratory infections and malnutrition, which are responsible for a high infant mortality rate, must take precedence over measures to control HAV [15]. Improved socioeconomic and sanitary conditions, including access to clean drinking water, reduce the risk of HAV-infection [16]. These improvements change the epidemiology of hepatitis A from high towards intermediate endemicity, with a shift in the age of infection towards older age groups and more clinically manifest disease [7], [17]. Our study comparing seroprevalence rates and risk of infection between 1995 and 2003 document this change for a deprived urban area in Nicaragua. Furthermore, it shows that consecutive serosurveys are a useful tool to assess the impact of public health measures on populations at risk.

An understanding of epidemiological patterns and shifts is essential for decisions on disease management and vaccination strategies [9]. Because endemicity may vary between regions and populations, estimates of epidemiological patterns are best generated based on samples from the same population at different time points. In our analysis we used both cross-sectional and longitudinal data on the seroprevalence of anti-HAV antibodies in residents of an area of León, Nicaragua with high hepatitis A endemicity [10], [18]. Using two cross-sectional surveys and assuming a constant and identical period effect for all age groups, we estimated a substantially lower risk of HAV infection in 2003 than in 1995. This decrease in infection risk is most likely due to incremental improvements in socio-economic standards and sanitary conditions, possibly paired with increased observation of hygiene in a population exposed to hepatitis A vaccine trials and other clinical studies for nearly one decade. The estimate for 1995 was in line with the results based on longitudinal data for children aged ≤3 years. An outlier was the risk in children aged 4, where the longitudinal 1995–96 data indicated a much lower risk compared with all other samples studied. This discrepancy might be explained by the fact that all children enrolled in the placebo arm of the trial had to be seronegative. The older children had to remain uninfected till age 4 and thus might have been at lower risk of HAV infection and therefore might not have been representative of all children of that age in the community.

In our 2003 population, seroprevalence rates were 100% for all age groups ≥18 years. Changes from high towards intermediate endemicity of hepatitis A will eventually lead to an increased proportion of susceptible adolescents and adults, especially in urban areas with higher socioeconomic standards [1], [17]. Moreover, infection rates are usually lower in urban than in rural areas [18]. It has been suggested that, in these circumstances, populations might benefit from vaccination against HAV [1], [19]. Routine vaccination of toddlers has been shown to be an effective approach to control hepatitis A in areas and countries shifting from high to intermediate or from intermediate to low endemicity [5], [6], [20], [21], [22], [23], [24].

A strength of the current analysis was the quantification of anti-HAV antibody levels assessed in the 2003 cross-sectional serosurvey. To our knowledge such quantitative data (Figure 3) have not been provided in surveys of seroprevalence, which typically report only percentages of seropositive individuals in a population. The large variation in GMC as well as the very high levels of anti-HAV antibodies were notable. Although the time points of the individual HAV infection in our population are not known, it can be inferred from the results that anti-HAV antibody levels may reach half a million mIU/mL during acute infections. It would be informative to compare our data with data from other areas and countries, but for this to be possible, absolute antibody values (mIU/ml) would need to be reported in addition to seroprevalence rates in similar studies.

In the 100% seroprevalent adult population there was a steady reduction in GMC with increasing age (Figure 3). In an area of high endemicity such as Léon, this observation of slowly declining antibody levels over the age range of 12 to 40 years provides indirect evidence of a solid infection-induced immunity. As individuals living in a high endemicity area will repeatedly be coming into contact with circulating HAV during adulthood - as documented in a recent survey for León (unpublished data) -, our findings suggest that such exposures have no boosting effect on antibody levels once protective immunity has been established by natural infection. In other words, no wild virus encounters may be needed to maintain a solid, life-long immune protection following natural infection. This finding from a high endemicity area is novel and seems to contradict the postulated role of natural boosting in maintaining long-term immunity for certain vaccine-preventable infections [25]. For example, the reduction in antibody concentrations found in Israeli toddlers soon after the introduction of universal mass vaccination against hepatitis A was interpreted as reflecting reduced natural boosting after the rapid reduction in circulating virus [6]. Of note, the so far successful single-dose toddler vaccination program initiated 2005 in Argentina [2], [26] indicates a similarly solid immunity after a single hepatitis A vaccination as shown here for natural infections, supporting current trends for simplified, i.e. single-dose hepatitis A vaccination schedules [1], [2], [27], [28].

Our study has several limitations. It is unclear whether our results can be generalized to other parts of the country with other HAV epidemiological patterns and socioeconomic and sanitary conditions. Further data will be required to guide decisions on appropriate local or national hepatitis A prevention strategies. Conducting repeated cross-sectional seroprevalence surveys in well targeted age-groups is a simple approach to obtain estimates of the incidence of new infections [12]–[14]. Estimates derived from such studies will only be accurate if the surveys are representative and the infection quite common. If the incidence is very low then other means of surveillance are more suited than sero-surveys. Accurate knowledge about current incidence levels is important to assess the success of prevention measures or to define the need for adjustments of prevention measures. Also, longitudinal 1995/96 data were only available for 117 children and no corresponding data were available from 2003. A further limitation of the analysis was that the risk of infection could not be estimated for children aged ≥7 years due to the high anti-HAV prevalence in this age group. If the shift in infection towards higher ages continues, lower anti-HAV prevalence in older children in the future may lead to a wider applicability of the method. Although not explicitly assessed in 1995, it is highly unlikely that any of the children screened for the field efficacy trial [10] had received hepatitis A vaccine beforehand as no such vaccine was licensed in Nicaragua at the time. The use of two different ELISA's with different, but WHO standardised cut-off levels for seropositivity in 1995 and 2003 reflects the objectives of the two studies, i.e. screening for seronegativity and quantitative measurement of antibody levels, and should not have introduced any bias.

In conclusion, the conduct of repeated cross-sectional surveys providing estimates of age-specific HAV antibody prevalence in countries of intermediate or high hepatitis A endemicity can be used to estimate the force of infection in a given population. Conducted over longer time periods, this approach would find potential use to monitor the impact of population-based public health interventions, such as provision of safe drinking water or the introduction of universal mass vaccination against hepatitis A in low-to-middle income countries. Furthermore it can be inferred from the quantitative anti-HAV antibody level results that contact with circulating HAV during adulthood seems not to have a boosting effect on antibody levels – as postulated in the past - once protective immunity has been established by natural infection.

## References

[1] World Health Organization (2012) WHO position paper on hepatitis A vaccines – June 2012. Wkly Epidemiol Rec 87: 261–276.22905367

[2] World Health Organization (2011) Immunization, Vaccines and Biologicals The Immunological Basis for Immunization Series. Module 18: Hepatitis A. Available: http://whqlibdoc.who.int/publications/2011/9789241501422_eng.pdf. Accessed 2013 Jul 8.

[3] Mohd Hanafiah K, Jacobsen KH, Wiersma ST (2011) Challenges to mapping the health risk of hepatitis A virus infection. Int J Health Geogr 10: 57. 22008459 10.1186/1476-072X-10-57 PMC3210090

[4] Bell BP, Shapiro CN, Alter MJ, Moyer LA, Judson FN, et al (1998) The diverse patterns of hepatitis A epidemiology in the United States - implications for vaccination strategies. J Infect Dis 178: 1579–1584. 9815207 10.1086/314518

[5] Dagan R, Leventhal A, Anis E, Slater P, Ashur Y, et al (2005) Incidence of hepatitis A in Israel following universal immunization of toddlers. JAMA 294: 202–210. 16014594 10.1001/jama.294.2.202

[6] Barkai G, Belmaker I, Givon-Lavi N, Dagan R (2009) The effect of universal toddlers-only hepatitis A virus vaccination program on seropositivity rate in unvaccinated toddlers: evidence for reduced virus circulation in the community. Pediatr Infect Dis J 28: 391–393. 19295466 10.1097/INF.0b013e318190655c

[7] Jacobsen KH, Koopman JS (2005) The effects of socioeconomic development on worldwide hepatitis A virus seroprevalence patterns. Int J Epidemiol 34: 600–609. 15831565 10.1093/ije/dyi062

[8] André FE (2006) Universal mass vaccination against hepatitis A. Curr Top Microbiol Immunol 304: 95–114. 16989266 10.1007/3-540-36583-4_6

[9] de Almeida LM, Amaku M, Azevedo RS, Cairncross S, Massad E (2002) The intensity of transmission of hepatitis A and heterogeneities in socio-environmental risk factors in Rio de Janeiro, Brazil. Trans R Soc Trop Med Hyg 96: 605–610. 12625132 10.1016/s0035-9203(02)90325-1

[10] Mayorga Perez O, Herzog C, Zellmeyer M, Loaisiga A, Frösner G, et al (2003) Efficacy of virosome hepatitis A vaccine in young children in Nicaragua: randomized placebo-controlled trial. J Infect Dis 188: 671–677. 12934183 10.1086/377309

[11] Brinkhof MWG, Mayorga O, Bock J, Heininger U, Herzog C (2013) Kinetics of maternally acquired anti-hepatitis A antibodies: prediction of waning based on maternal or cord blood antibody levels. Vaccine 31: 1490–1495. 23328312 10.1016/j.vaccine.2013.01.011

[12] Keiding N (1991) Age-specific Incidence and Prevalence: a Statistical Perspective. J R Statist Soc 154: 371–412.

[13] Laubereau B, Zwahlen M, Neuenschwander B, Heininger U, Schaad UB, et al (2000) Herpes simplex virus type 1 and 2 in Switzerland. Schweiz Med Wochenschr 130: 143–150. 10701231

[14] Ades AE, Nokes DJ (1993) Modeling age- and time-specific incidence from seroprevalence: toxoplasmosis. Am J Epidemiol 137: 1022–1034. 8317447 10.1093/oxfordjournals.aje.a116758

[15] Ministerio de Salud MINSA (2007) Indicadores Básicos en Salud. Available: http://www.minsa.gob.ni/index.php?option=com_remository&Itemid=52&func=fileinfo&id=5285. Accessed 2013 Jul 8.

[16] Jacobsen KH, Koopman JS (2004) Declining hepatitis A seroprevalence: a global review and analysis. Epidemiol Infect 132: 1005–1022. 15635957 10.1017/s0950268804002857 PMC2870191

[17] Tanaka J (2000) Hepatitis A shifting epidemiology in Latin America. Vaccine 18 (Suppl 1) S57–S60. 10683550 10.1016/s0264-410x(99)00466-1

[18] Perez OM, Morales W, Paniagua M, Strannegard O (1996) Prevalence of antibodies to hepatitis A, B, C, and E viruses in a healthy population in Leon, Nicaragua. Am J Trop Med Hyg 55: 17–21. 8702016

[19] Wasley A, Fiore A, Bell BP (2006) Hepatitis A in the era of vaccination. Epidemiol Rev 28: 101–111. 16775039 10.1093/epirev/mxj012

[20] van Damme P, Van Herck K (2005) Effect of hepatitis A vaccination programs. JAMA 294: 246–248. 16014600 10.1001/jama.294.2.246

[21] Quezada A, Baron-Papillon F, Coudeville L, Maggi L (2008) Universal vaccination of children against hepatitis A in Chile: a cost-effectiveness study. Rev Panam Salud Publica 23: 303–312. 18510790 10.1590/s1020-49892008000500002

[22] Lopez E, Debbag R, Coudeville L, Baron-Papillon F, Armoni J (2007) The cost-effectiveness of universal vaccination of children against hepatitis A in Argentina: results of a dynamic health-economic analysis. J Gastroenterol 42: 152–160. 17351805 10.1007/s00535-006-1984-x

[23] Victor JC, Surdina TY, Suleimenova SZ, Favorov MO, Bell BP, et al (2006) Person-to-person transmission of hepatitis A virus in an urban area of intermediate endemicity: implications for vaccination strategies. Am J Epidemiol 163: 204–210. 16339053 10.1093/aje/kwj029

[24] Sartori AM, de Soárez PC, Novaes HM, Amaku M, de Azevedo RS, et al (2012) Cost-effectiveness analysis of universal childhood hepatitis A vaccination in Brazil: regional analyses according to the endemic context. Vaccine 30: 7489–7497. 23107593 10.1016/j.vaccine.2012.10.056

[25] Pichichero ME (2009) Booster vaccinations: can immunologic memory outpace disease pathogenesis? Pediatrics 124: 1633–1641. 19933727 10.1542/peds.2008-3645

[26] Vizzotti C, González J, Gentile A, Rearte A, Ramonet M, et al (2014) Impact of the single dose immunization strategy against hepatitis A in Argentina. Ped Infect Dis J 33: 84–88. 10.1097/INF.0000000000000042 24352191

[27] World Health Organization (2012) Meeting of the Strategic Advisory Group of Experts on immunization, April 2012 - conclusions and recommendations. Wkly Epid Rec 87: 201–216. 24340402

[28] Ott JJ, Wiersma ST (2013) Single-dose administration of inactivated hepatitis A vaccination in the context of hepatitis A vaccine recommendations. Int J Infect Dis 17: e939–e944. 23791857 10.1016/j.ijid.2013.04.012

